# Efficacy of a multimodal lifestyle intervention (The Lift Project) for improving the mental health of individuals with an affective mood disorder living in South Africa

**DOI:** 10.3389/fpsyg.2023.1127068

**Published:** 2023-01-25

**Authors:** Amanda Oakes-Cornellissen, Darren Morton, Paul Rankin, Melanie Renfrew

**Affiliations:** Lifestyle Medicine and Health Research Centre, Avondale University, Cooranbong, NSW, Australia

**Keywords:** lifestyle, intervention – behavioral, depression, anxiety, stress, affective disorder, lifestyle medicine, positive psychology

## Abstract

**Background:**

Affective disorders are becoming more pervasive worldwide, including in Southern Africa, where treating patients with these conditions is challenging due to social and financial constraints. A variety of non-pharmacological approaches including lifestyle medicine (e.g., exercise, nutrition, sleep) and positive psychology practices (e.g., gratitude, service), are effective for treating mental health (MH) conditions.

**Methods:**

Twenty-six individuals from South Africa with a diagnosed MH condition participated in a 10-week multimodal intervention incorporating a diverse range of non-pharmacological strategies for improving MH. MH metrics were assessed pre-and post-intervention, including general MH, vitality/energy (VIT), depression, anxiety, stress, and satisfaction with life. MH and VIT were also measured weekly.

**Results:**

Improvements were observed in all mental metrics from pre-to post-intervention: MH (59%, *p* < 0.001, Cohen’s *D* = 1.36), VIT (110%, *p* < 0.001, Cohen’s *D* = 1.71), depression (−46%, *p* < 0.001, Cohen’s *D* = −1.06), anxiety (−48%, *p* < 0.001, Cohen’s *D* = −1.21), stress (−36%, *p* < 0.001, Cohen’s *D* = −1.08) and life satisfaction (23%, *p* < 0.001, Cohen’s *D* = 0.66). Significant improvements in MH and VIT were observed after only 1 week of the intervention and progressively increased until the seventh week, after which further improvements were not statistically significant.

**Conclusion:**

The findings of this cohort study indicate that a multimodal intervention that incorporates lifestyle and positive psychology practices may benefit individuals living with an affective disorder. Non-pharmacological, multimodal interventions might offer a stigma-free way of providing MH promotion and treatment at a population level.

## 1. Introduction

The most common psychological conditions reported globally are depression and anxiety (Albert, 2015; Laborde-Lahoz et al., 2015). According to the World Health Organization (WHO) World Mental Health Survey, South Africa ranks among the highest mid-to-low-income countries in the lifetime prevalence of Common Mental Disease (CMD; Stein et al., 2008). It has been postulated that South Africa’s hostile history, which resulted in social inequality, conflict, and trauma, has contributed to the high prevalence of psychological distress (Das-Munshi et al., 2016; Harriman et al., 2022). Studies indicate that within South Africa, affective disorders are especially common in low socioeconomic areas (Mungai and Bayat, 2019) and in communities with high levels of social dysfunction, crime rates, and gangsterism (Tomita et al., 2015).

Despite the high prevalence of affective disorders in South Africa, mental health (MH) conditions are underdiagnosed and underreported, leading to poor health outcomes for those living with untreated mental illnesses (Meyer et al., 2019). Indeed, at an international level, expenditure on MH services in many countries is estimated at 5% or less of overall healthcare budgets (Gupta et al., 2016). With the immense financial strain that South Africa’s public health sector is experiencing, clients with CMD often fail to receive the necessary care, or any care at all (Kagee, 2008). Hence, novel approaches to addressing the MH burden are required.

The treatment of affective disorders is increasingly predicated on pharmacological protocols, yet the prevalence of MH conditions continues to rise (Mojtabai and Jorm, 2015; Jorm et al., 2017), raising questions regarding the efficacy of anti-depressant medication for curbing the escalating rates of mental distress (Khan and Brown, 2015). Further, while pharmacological approaches have benefits, they may complicate the disease profile with unpleasant side effects (Huhn et al., 2014) such as “feeling emotionally numb,” detached from reality, drowsiness, sexual dysfunction, and suicidal ideation, to name a few (Read and Williams, 2018).

There is increasing interest in the use of non-pharmacological approaches for improving MH (Manger, 2019). Indeed, there is strong evidence showing the MH benefits of interventions that promote healthy lifestyle behaviors such as exercise (Josefsson et al., 2014; Keating et al., 2018), healthy eating (Jacka et al., 2017; Owen and Corfe, 2017; Firth et al., 2020); sleep (Freeman et al., 2020; Scott et al., 2021), and exposure to nature (Taniguchi et al., 2022). In addition, positive psychology practices have been shown to improve MH (Seligman and Csikszentmihalyi, 2014; Carr et al., 2021), including expressing gratitude (Wong et al., 2018; Waters et al., 2022); identifying and using one’s signature strengths (Macfarlane, 2019); and engaging in service activities (Walsh, 2011). Notably, the implementation of lifestyle and positive psychology therapies comes with minimal to no deleterious side effects, which highlights their utility (Marx et al., 2022). However, while the benefits of these lifestyle and positive psychology strategies have been demonstrated, they are often applied as single modality interventions. The emerging discipline of “Lifestyle Psychiatry” is calling for a broader, multimodal approach (Noordsy, 2019).

A multimodal intervention that has been shown to improve participants’ MH and wellbeing is “The Lift Project” (Morton et al., 2020; Przybylko et al., 2021b,c; Renfrew et al., 2021b). The Lift Project is a positively oriented 10-week education program that experientially engages participants in wellbeing-enhancing strategies from the disciplines of lifestyle medicine and positive psychology. The Lift Project was initially designed as a mental wellbeing program for generally healthy cohorts, and numerous studies, including randomized controlled trials, have reported positive outcomes among this target population (Przybylko et al., 2021a; Renfrew et al., 2021a). However, stratified analyses have indicated that while the intervention adopts a positive orientation, participants with the lowest baseline MH metrics may benefit the most (Morton et al., 2020). This may suggest that promoting the positive may negate the negative, even among individuals with poor MH.

This study aimed to examine the potential benefits of The Lift Project intervention for adults with a diagnosed affective mood disorder living in a community in South Africa that exhibits high levels of mental distress. If efficacious, non-pharmacological interventions like The Lift Project that are positively oriented and adopt a multimodal approach may provide a useful tool for supporting individuals who suffer from affective disorders, and thereby offer a helpful solution for addressing the MH burden.

## 2. Methodology

### 2.1. Study design

The objective of this study was to pilot-test the efficacy of a 10-week intervention for improving quantitative MH metrics among adults living with a diagnosed affective mood disorder. Hence, a single-arm, pre-post cohort design was selected as a precursor to a larger randomized controlled trial, pending the study showing beneficial outcomes. The 10-week intervention commenced in August and concluded in October 2022. Ethical clearance for the study was obtained by the Avondale University Human Ethics Committee (approval no: ETH.2022.009).

### 2.2. Study participants

The study was promoted to patients from a General Practice in Kathu, a small mining town in the Northern Cape of South Africa with a population of approximately 14,000 residents as reported at the 2016 census. This location was selected as the community is underserved and has a high prevalence of mental distress. It was rationalized that if benefits were observed in this challenging context, it might represent a novel approach for addressing mental distress in other high-need, poorly funded communities.

The inclusion criteria for the study were:

1. Males and females between the ages of 18 and 65 years. Affective disorders in South Africa are prevalent among both males and females and within this broad age range and hence, the intervention was made available to all adults with a MH diagnosis (as defined below).
2. Currently taking either anti-depressant, anxiolytic, or mood stabilizing medication for an affective mood disorder.
3. A score of ≤56 on the MH scale or ≤45 on the vitality scale (VIT) of the SF-36 health assessment (Ware and Sherbourne, 1992). The literature indicates that the MH is a good predictor of MH disorders, including depression, anxiety, and affective disorders generally (Berwick et al., 1991). A MH score ≤56 is considered indicative of major depression. A VIT score of ≤45 represents clinically significant fatigue (Donovan et al., 2008).

The exclusion criteria were:

1. Pregnant or lactating.
2. Scheduled to be taken off prescribed medication for their relevant mood disorder(s) during the scheduled 10-week period of the intervention.

Thirty-one participants applied to participate in the study. The data for five individuals were excluded from the study due to their MH or VIT scores exceeding those specified in the inclusion criteria above, however, these individuals were allowed to participate in the intervention. Three participants exited the study within the first week as they did not want to participate in the intervention. The remaining 23 participants (mean age = 41.2 ± 10.8 years, 19 females/4 males) constituted the study cohort.

### 2.3. “The Lift Project” intervention

The intervention was conducted over 10 weeks and explored evidence-based, non-pharmacological strategies for increasing hedonic and eudemonic wellbeing. Each week, the subjects participated in a 60-min group session hosted at a local General Practice clinic. Three group sessions were offered each week for the participants to choose from (Monday, Tuesday, or Wednesday evenings), with a maximum of ten participants in a group. Participants who were not able to attend either of the three sessions for that week were given the option to attend a catch-up session, but these remained small as most participants were able to attend one of the three scheduled sessions. All 23 participants successfully completed all of the 10 lessons of the intervention over the 10 weeks and hence received and applied all the content as set out in Table 1. Five participants attended the intervention *via* Zoom due to an inability to attend in person. All the group sessions were conducted by the same facilitator who was trained and resourced to deliver the program through an online portal that included: an overview of the program and its rationale, weekly lesson plans, and ideas for conducting the program. The facilitator was also given full access to the program to become familiar with the content of each lesson.

**Table 1:** Overview of The Lift Project intervention.

Week	Topic
1	Title: Speak positively. Synopsis: Introduction to the emotional brain (the Limbic system) and the influence of language on emotion. Challenges: Compliment others; memorize inspirational literature.
2	Title: Move dynamically. Synopsis: The influence of physical activity on mental health. Challenges: Daily step challenge; engage in resistance exercises.
3	Title: Immerse in an uplifting physical environment. Synopsis: Environmental influences on mental health. Challenges: 30 min daily outdoor time; view the sunrise.
4	Title: Immerse in an uplifting social environment. Synopsis: The impact of relationships on mental wellbeing and strategies for nurturing positive relationships. Challenges: Engage the love languages; make a new friend in person or forgive a friend/family member.
5	Title: Look to the positive. Synopsis: The mental health benefits of focusing on the positive in the past, present and future. Challenges: Daily journal of “what went well;” perform the “Gratitude Visit.”
6	Title: Eat nutritiously. Synopsis: The influence of a whole food, plant-based diet on mental health. Challenges: 8 daily serves of plant foods; prepare and share a healthy plant-based meal.
7	Title: Rest – sleep well. Synopsis: The influence of good sleep on mental health. Challenges: Restrict screen usage before bed; spend an evening by firelight.
8	Title: Rest - destress. Synopsis: Positive strategies for managing stress. Challenges: 10 min of daily mindfulness; take a guilt free day off.
9	Title: Serve. Synopsis: The value of service on mental health and how to serve using signature strengths. Challenges: Random acts of kindness; recognize and apply unique signature strengths for good.
10	Title: Flourish Synopsis: Highlighting the PERMA framework for enhancing overall wellbeing (positive emotion, engagement, relationships, meaning, and achievement). Challenges: Engage in activities that are enjoyable; set goals, and work toward achieving them.

The intervention utilized a pedagogical framework abbreviated as LETS—Learn, Experience, Think, and Share. At each session, the participants viewed an educational video that presented the evidence-base for the topic (i.e., “Learn”). The video presentations were approximately 15 min in duration and were presented in a positive tone. Arising from the video presentation, the participants were issued with challenges for the upcoming week that involved them personally putting the learnings into practice (i.e., “Experience”). These were issued as “challenge by choice,” but with the reminder that the more they engaged with challenges the more benefit they would likely experience. The participants were then encouraged to reflect on what they were learning and experiencing (i.e., “Think”) by completing a workbook/journal that was provided to them. The workbook reinforced the key messages of the lessons and provided the participants with space to reflect on what they were learning and experiencing. Finally, the participants were encouraged to “Share” their learnings and experiences through group discussion activities, as well as share with others from within their sphere of influence.

An overview of the topics covered in the intervention and the associated challenges are shown in Table 1.

### 2.4. Measurements

The participants completed a health and wellbeing questionnaire at the beginning and end of the 10-week intervention. The questionnaire took approximately 15–20 min to complete and used validated instruments, explained below, to assess: General MH, vitality/energy (VIT), Depression, Anxiety, Stress, and Satisfaction with Life. In addition, MH and VIT were assessed at the beginning of each weekly lesson, which took approximately 2 min to complete.

#### 2.4.1. MH and vitality (VIT)

Two subscales from the 36-item Short Form Health Survey (Contopoulos-Ioannidis et al., 2009) were used in this study to assess general MH (5 items) and vitality/energy (VIT, 4 items) pre-and post-intervention as well as every week throughout the 10-week intervention.

Each item in the MH and vitality subscales has six response options ranging from “All of the time” to “None of the time.” The standard procedure was used to calculate a score between 0 to 100 for MH and VIT (Ware et al., 2000), with higher scores indicating better MH and vitality. Studies have reported a Cronbach alpha of 0.90 for the MH scale and 0.87 for the VIT scale, indicating good internal consistency (Jenkinson et al., 1994). In the present study, Cronbach alphas of 0.84 and 0.83 were recorded for MH and vitality, respectively.

#### 2.4.2. Depression, anxiety, and stress scale

The 21-item Depression, Anxiety, and Stress Scale (DASS-21), which is appropriate for use in both clinical and nonclinical populations (Osman et al., 2012), was administered pre-and post-intervention to assess depression, anxiety, and stress (7 items per subscale). The 21-items of the DASS-21 are assessed on a 4-point Likert scale, asking respondents to rank their symptoms of depression, anxiety, and stress as “Never,” “Sometimes,” “Often,” and “Almost always.” Scores for each of the three domains were converted to a total score out of 100, with higher scores indicating worsening symptoms. Good internal consistency has been reported for the three domains of the DASS-21, with Cronbach alphas ranging from 0.76 to 0.91 (Le et al., 2017). The present study observed a Cronbach alpha of 0.85 for depression, 0.75 for anxiety and 0.83 for stress.

#### 2.4.3. Satisfaction with life scale

The 5-item SWLS is extensively used as a measure of overall life satisfaction (Pavot et al., 1991). It involves five questions measured on a 6-point Likert scale with responses ranging from “Strongly disagree” to “Strongly agree.” The Cronbach alpha was 0.85, which is higher than that reported (Vassar, 2008) in a meta-analysis of the instrument (Cronbach alpha = 0.78).

### 2.5. Sample size calculation and statistical analyses

The required sample size was calculated using data from previous studies that have utilized the same intervention (Morton et al., 2020) and was based on the following assumptions: a change in MH scores of 20%; 80% power and significance level of 0.05 (95% confidence interval); a moderate effect size (i.e., 0.5); an attrition rate of 30%.

The data were entered and screened using Microsoft Excel (version 16) before being imported into IBM SPSS statistical software package (version 28) for analysis. Descriptive statistics, including mean and standard deviation, were used to present the data. Paired *t-*tests were used to determine differences in the outcome measures from baseline to post-intervention. As six outcome measures were analyzed, a Bonferroni correction was applied, resulting in the adoption of a level of significance of 0.008 (i.e., 0.05/6). Effect size (Cohen’s D) was calculated as the mean change in the outcome measure divided by the standard deviation of the subjects’ mean difference scores. Repeated-measures ANOVA with *post-hoc* analyses were used to examine changes in the weekly MH and VIT measures.

## 3. Results

As shown in Table 2, significant improvements were observed in all outcome measures (at the 0.008 level), with large effect sizes. Based on scores reported by the participants at the conclusion of the study, approximately two-thirds (i.e., 15 of the 23) would not have met the study inclusion criteria (i.e., a MH score of ≤56 or a VIT score of ≤45).

**Table 2:** Changes in the outcome measures from baseline to post-intervention.

Measure	Pre-test	Post-test	Change	% Change	*t* Statistic	*p* value	Cohen’s *D*
	Mean (SD)	Mean (SD)					
Mental health (MH)	43.6 (22.1)	69.4 (15.9)	25.9	59.3	8.39	<0.001	1.36
Vitality/energy (VIT)	29.8 (21.8)	62.9 (16.8)	33.0	110.8	8.01	<0.001	1.71
Depression	51.7 (26.8)	27.8 (18.4)	−23.9	−46.3	−6.75	<0.001	−1.06
Anxiety	44.3 (19.0)	23.1 (16.1)	−21.2	−47.9	−5.52	<0.001	−1.21
Stress	55.2 (20.9)	35.5 (15.5)	−19.7	−35.7	−5.00	<0.001	−1.08
Life satisfaction	55.1 (19.8)	68.3 (20.0)	13.2	23.9	3.37	0.002	0.66

**Figure 1:**
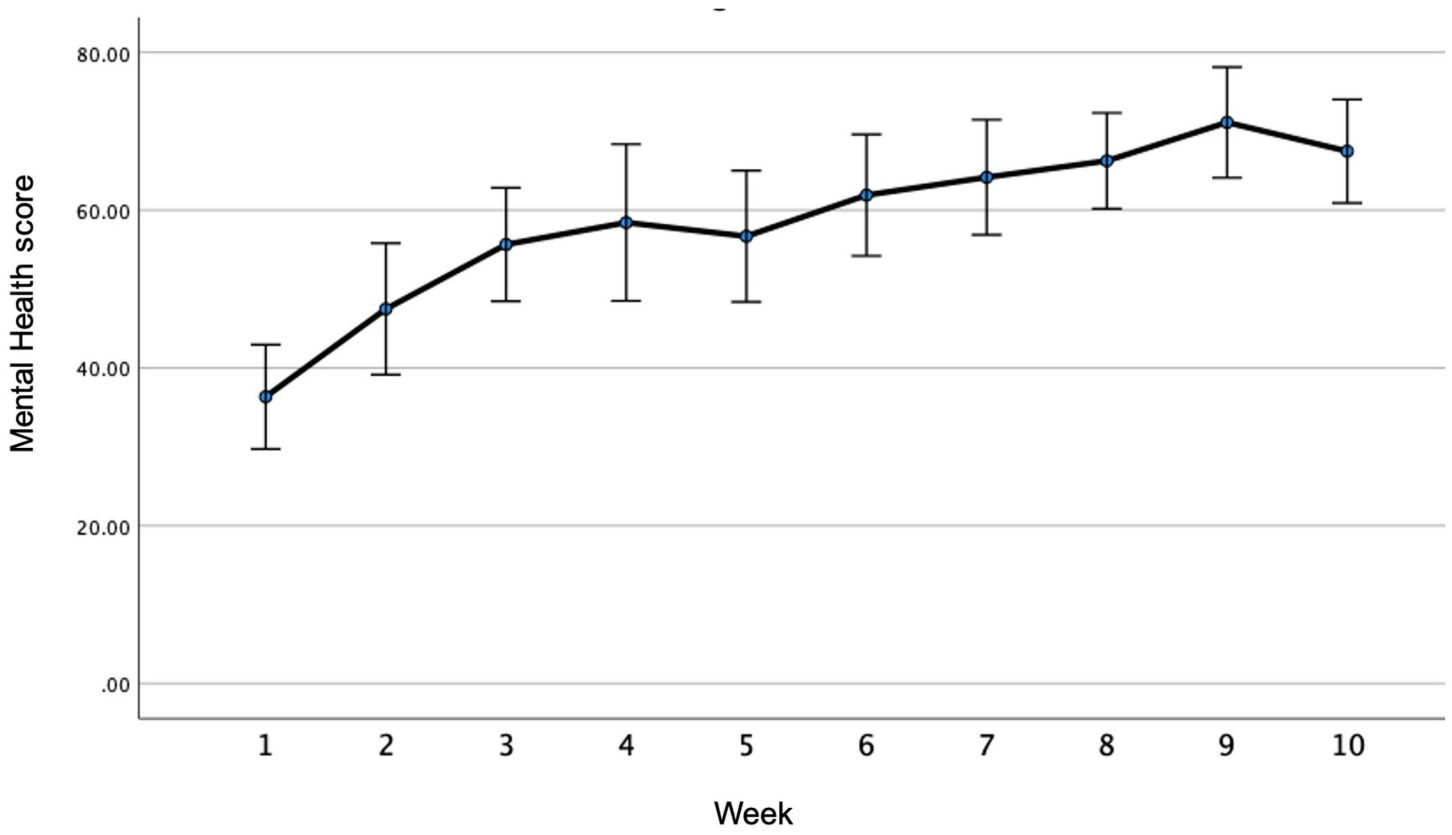
Weekly mental health (MH) scores throughtout the intervention.

**Figure 2:**
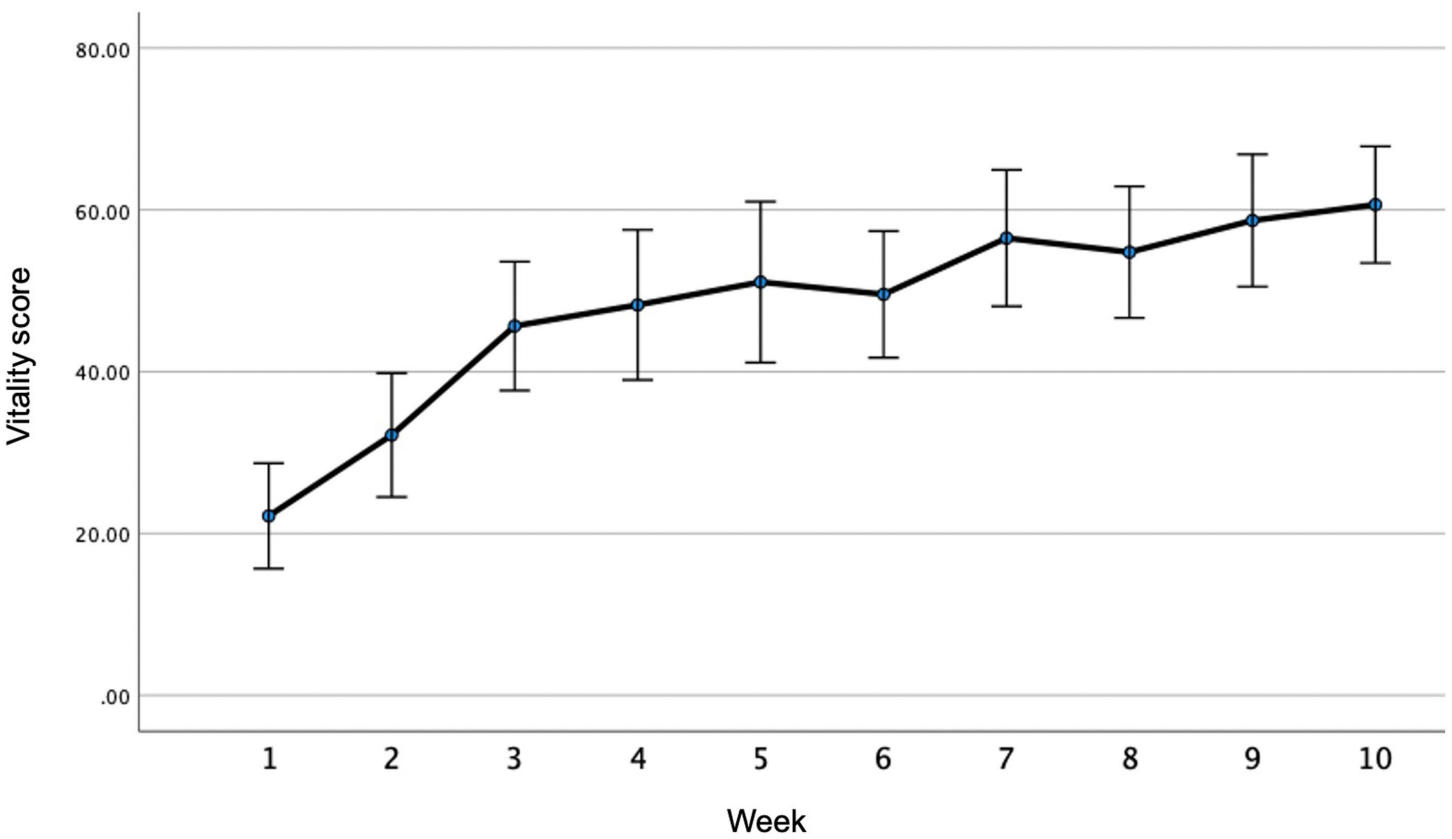
Weekly vitality/energy (VIT) scores throughtout the intervention.

The mean changes in the weekly MH and VIT scores are illustrated in Figures 1, 2, respectively. The ANOVA indicated significant changes over time for both MH (F ratio = 15.4, *p* ≤ 0.001, Partial Eta Squared = 0.412) and VIT (F ratio = 20.5, *p* ≤ 0.001, Partial Eta Squared = 0.483). *Post-hoc* analyses indicated that significant improvements were reported after only 1 week in both MH (*p* = 0.005) and VIT (*p* = 0.015), after which the reported MH and VIT scores were significantly higher than baseline at the <0.001 level for all subsequent weeks. As can be observed in Figures 1, 2, there was a trend for incremental improvement in the MH and VIT scores over the 10 weeks of the intervention.

Two participants made changes to their prescriptions over the duration of the study. Thyroid medication was added to one participant’s medication list in week 9 of the intervention and another participant had their anti-depressant medication reduced to half during week 4.

## 4. Discussion

The findings of this pilot study suggest that positively oriented MH interventions that incorporate an array of lifestyle medicine and positive psychology strategies may support and improve the MH of individuals with diagnosed affective disorders. Previous studies have demonstrated that The Lift Project intervention used in this study is efficacious and acceptable among healthy cohorts, but the outcomes of this study suggest that the intervention might benefit people across the MH spectrum. These findings may have important implications as they suggest that interventions similar in design to that used in the current study might offer a stigma-free way of providing MH promotion and treatment at a population level, using a universal prevention approach.

The effect sizes observed in this study were substantially larger than those observed in other studies that have utilized the same intervention among relatively healthy cohorts. For example, in a randomized controlled trial involving 425 general community members from Australasia, effect sizes of 0.42 and 0.45 were observed for change in MH and VIT, respectively (Przybylko et al., 2021a). The very large effect sizes observed in the present study (i.e., >1.0) are encouraging, although it is recognized that the individuals have more scope of improvement when starting from a lower baseline. Indeed, in a study of The Lift Project intervention among College students, the greatest improvements were observed among those with the poorest baseline MH scores (Morton et al., 2020).

This is the first study of The Lift Project to document weekly changes in MH and VIT throughout the duration of the 10-week intervention. The observation that significant improvements in MH and VIT were recorded after only 1 week is notable, but the impetus for such rapid improvements warrants investigation. It is hypothesized that the act of engaging with an intervention might instill hope, which alone is mood-enhancing, independent of the “challenges” issued to the participants as part of the intervention. Indeed, hope is an important MH determinant (Van Gestel-Timmermans et al., 2010; Schrank et al., 2012). While further studies are required to ascertain the potential contributors to the observed rapid improvements in MH and VIT, these measures continued to increase incrementally throughout the duration of the intervention which suggests that the intervention did not merely instill “false hope,” but that the activities were beneficial.

While the week-by-week MH and VIT data showed a progressive upward trend over the 10 weeks of the intervention, further studies are required to confirm these observations and to determine causality. Understanding the time-course of potential improvements in MH metrics as well as “plateauing” may help inform the design of interventions with regard to optimal duration. Shorter interventions may be administered more cost-effectively, although interventions of longer durations might better support long-term behavior change. A study involving a lifestyle intervention targeting cardiovascular disease found that an 8 or 16 sessions intervention produced similar outcomes in the short-term (Morton et al., 2017), but longer interventions might better cement behavior change and result in more sustained benefits. Indeed, there is a need to further investigate the relative merits of shorter and longer interventions, especially with regard to long-term benefits. While the current pilot study only examined pre-to post-intervention changes, a randomized controlled trial using the same intervention observed sustained benefits at 3-month follow-up (Przybylko et al., 2021a). Longer-term follow-up should be included in future studies.

### 4.1. Limitations

There are several limitations of this study. Firstly, several factors affect the generalizability of the findings. As the study involved a relatively small cohort it was not possible to explore the influence of covariates such as age, sex, and group. Further, the participants were self-selecting and therefore may have presented to the study with a high readiness for change. Certainly, the low attrition rate (<10%) would suggest that the participants in the study were prepared to take action, as framed by the Transtheoretical Model of Behavior Change (Prochaska et al., 2009). Further affecting the generalizability of the findings is the strong female participation bias, which is commonly observed in lifestyle interventions (Pot et al., 2019; Przybylko et al., 2021b). A second limitation relates to the absence of a control group. As a result, it is not possible to determine the influence of confounders, such as seasonal variation, on the outcomes observed in the study. Future studies should also capture details about the extent to which the participants engage in the weekly challenges to investigate the association between adherence to the intervention and the benefits obtained. Certainly, the findings of this pilot study warrant a rigorous clinical trial.

## 5. Conclusion

The findings of this pilot study indicate that a positively oriented, multimodal intervention that incorporates lifestyle medicine and positive psychology strategies may benefit individuals living with an affective disorder. Given that interventions like The Lift Project have also shown to be efficacious among non-clinical cohorts, it may support the use of such interventions at a population level as a universal MH prevention strategy.

## Data availability statement

The datasets presented in this article are not readily available because Ethical clearance to share the data set has not been granted. Requests to access the datasets should be directed to darren.morton@avondale.edu.au.

## Ethics statement

The studies involving human participants were reviewed and approved by Avondale University Human Research Ethics committee. The patients/participants provided their written informed consent to participate in this study.

## Author contributions

AO-C was responsible for conceiving the study, conducting the intervention, data collection and collation, and writing the paper. DM led the development of the intervention and assisted with data analyses. DM, MR, and PR contributed to the study design and provided editorial input on the paper. All authors contributed to the article and approved the submitted version.

## Funding

Publication costs were provided by the Lifestyle Medicine and Health Research Centre at Avondale University.

## Conflict of interest

DM is the founding director of a profit-for-purpose trust that owns the intervention used in this study. He has received no financial reimbursement.

The remaining authors declare that the research was conducted in the absence of any commercial or financial relationships that could be construed as a potential conflict of interest.

## Publisher’s note

All claims expressed in this article are solely those of the authors and do not necessarily represent those of their affiliated organizations, or those of the publisher, the editors and the reviewers. Any product that may be evaluated in this article, or claim that may be made by its manufacturer, is not guaranteed or endorsed by the publisher.
